# Spatio-Temporal Pattern and Influencing Factors of Hemorrhagic Fever with Renal Syndrome (HFRS) in Hubei Province (China) between 2005 and 2014

**DOI:** 10.1371/journal.pone.0167836

**Published:** 2016-12-28

**Authors:** Liang Ge, Youlin Zhao, Kui Zhou, Xiangming Mu, Haibo Yu, Yongfeng Wang, Ning Wang, Hong Fan, Liqiang Guo, XiXiang Huo

**Affiliations:** 1 State Key Laboratory of Information Engineering in Surveying, Mapping and Remote Sensing, Wuhan University, Wuhan city, Hubei Province, PR China; 2 Tianjin Institute of Surveying and Mapping, Tianjin city, PR China; 3 Business School of Hohai University, Nanjing city, Jiangsu Province, PR China; 4 School of Information Studies in University of Wisconsin-Milwaukee 2025 E Newpot Ave #NWQB, Milwaukee, WI, United States of America; 5 First Crust Deformation Monitoring and Application Center, China Earthquake administration, Tianjin city, PR China; 6 Hubei Provincial Center for Disease Control and Prevention, Wuhan, China; The Scripps Research Institute, UNITED STATES

## Abstract

Hemorrhagic Fever with Renal Syndrome (HFRS) is considered as a globally distributed infectious disease, which results in many deaths annually in Hubei Province, China. The outbreak of HFRS is usually characterized with spatio-temporal heterogeneity and is seasonally distributed. Further, it might also be impacted by the influencing factors such as socio-economic and geographical environment. To better understand and predict the outbreak of HFRS in the Hubei Province, the spatio-temporal pattern and influencing factors were investigated in this study. Moran’s *I* Index value was adopted in spatial global autocorrelation analysis to identify the overall spatio-temporal pattern of HFRS outbreak. Kulldorff scan statistical analysis was performed to further identify the changing trends of the clustering patterns of HFRS outbreak. Spearman's rank correlation analysis was used to explore the possible influencing factors on HFRS epidemics such as climate and geographic. The results demonstrated that HFRS outbreak in Hubei Province decreased from 2005 to 2012 in general while increasing slightly from 2012 to 2014. The spatial and temporal scan statistical analysis indicated that HFRS epidemic was temporally clustered in summer and autumn from 2005 to 2014 except 2008 and 2011. The seasonal epidemic pattern of HFRS in Hubei Province was characterized by a bimodal pattern (March to May and September to November) while peaks often occurring in the spring time. SEOV-type HFRS was presumed to influence more on the total number of HFRS incidence than HTNV-type HFRS do. The average humidity and human population density were the main influencing factors during these years. HFRS outbreaks were more in plains than in other areas of Hubei Province. We did not find that whether the terrain of the wetland (water system) plays a significant role in the outbreak of HFRS incidence. With a better understanding of rodent infection rate, socio-economic status and ecological environment characteristics, this study may help to reduce the outbreak of HFRS disease.

## Introduction

In China, HFRS was mainly caused by two types of Hantaviruses, named Hantaan virus (HTNV) and Seoul virus (SEOV), each associated with a unique rodent host [1–3]. In China, the first case of HFRS was discovered in 1935. After that, data showed that the number of HFRS cases in China accounts for 90% of the globally reported cases over the past 20 years [4–7].

Generally, human activities and natural factors were related to the occurrence and epidemic of *hantaviruses* [8–10]. In Europe, in order to discover the regular pattern and feature of HFRS, hank vole dynamics, soil contamination dynamics and human contamination dynamics were used to explain the spatial variation of HFRS outbreak [11]. In China, some researchers used cluster analysis to study the relationships between the spatial distribution and the influencing factors of the HFRS outbreaks to explore the degree of clustering. Poisson regression analysis was performed by Zhang et al. to identify the HFRS transmission pattern in north-eastern China from year 1997 to 2007 [12]. Climate factors (e.g. monthly rainfall, relative humidity, and land surface temperature) were found to be the determinants to HFRS transmission in this research. Moran’s *I* spatial autocorrelation statistical and retrospective spatio-temporal clustering methods were used by Wu et al. to analyze the spatio-temporal distribution in Liaoning Province from 1988 to 2001. They demonstrated that the outbreak of HFRS had homogeneous spatio-temporal characteristics [5]. Lin et al performed Spatial smoothing and Martin Kulldorff’s spatial scan test to study the spatial distribution and variation of the HFRS outbreak in Liaoning Province between 2000 and 2005 [4]. They found that the clusters of HFRS cases were consistently influencing by humidity and the amount of forestation. In another study, ARIMA model and historical time series data were utilized by Liu et al. to simulate the temporal distribution tendency of HFRS in China from 1975 to 2008. The results demonstrated that ARIMA model had a good feasibility to forecast the HFRS outbreak [13].

Although a variety of methods have been implemented to reduce the occurrence of HFRS, the HFRS cases were still more than 20,000 annually in China from 1980 to 2009 according to the report [12]. In Hubei Province, the number of HFRS cases reached 23,943 cases in 1983 [12,14,15], and get 104,467 cases in total between 1980 and 2009 [11,16,17]. The relationships between natural environment and number of HFRS outbreaks were explored in Russia and Korea in the 1990s [18]. Since 2000, for the outbreak of HFRS in northeast China, most researches began to focus on a short term (five years or four years) relationships between dynamic spatial distribution and possible influencing factors [12,15]. In this study, we will explore a ten year appeared relationships between dynamic spatial distribution and possible influencing factors in Hubei Province.

Liu et al. found that temporal and geographic factors both played important roles on the outbreaks of HFRS in central-south China between 2000 and 2009 [19]. Zhang et al. studied the spatio-temporal distribution pattern of HFRS in Hubei Province over 1980–2009, they found that the number of HFRS cases was decreasing in general during this period [14]. Through the data from the experiment analysis in Hubei Province, this study will explore the distribution tendencies of clustering areas and the dominated influencing factors on a year-by-year base. The significant of this study is to characterize the overall spreading tendency and moving patterns of HFRS in recent years in Hubei Province.

## Research Methodology

### Study area and data source

**Fig 1:**
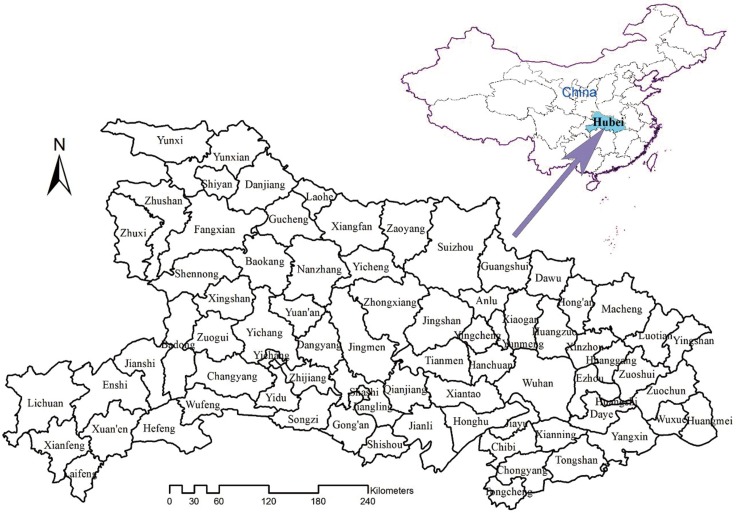
The location of Hubei Province in China. The highlighted block in the above map indicates its location. The underneath map indicates the county boundary of Hubei Province.

Fig 1 displays the location of Hubei Province in China. Hubei Province is located in the central south part of China. In the middle of the Yangtze River with an area of 186,000 square kilometres, it is located at 108'21"-116'07" east longitude and 29'05"-33'20" north latitude. The population was about 59.37 million in 2005. Up to 2013, it had one autonomous prefecture, 12 prefecture-level cities, 24 county-level cities, 38 counties, two autonomous counties and one forest district. Administrative region of counties in Hubei Province have changed for several times. In order to organize the data effectively during data processing, 76 counties have been integrated according to the latest Chinese Administrative Coding Rules.

Four data sources were used in this study. First, the number of HFRS cases of 76 counties in Hubei Province was provided by Hubei Provincial Centre for Disease Control and Prevention (CDC) between 2005 and 2014. Secondly, human population data was provided by Hubei Provincial Bureau of Statistics based on the 2009 censuses. Thirdly, the map at a scale of 1:4000, 000 was obtained by National Geomatics Centre of China. Fourthly, climatic data from 2005 to 2014 was provided partly by Hubei Provincial Meteorological Bureau and partly from online open sources. These four sources of data were stored in the geographical information database and attribute database. Geographical information database was linked to administrative region by using ArcGIS 10.2. The extent contour and central points for each county were also stored in the geographical information database. Data fields included name, centre point coordinates, county code and etc. Attribute database consists of HFRS cases data per month and per year for each county, demographics and climate data. Data fields for attributes data included county code, average temperature value, average humidity value, average rainfall value, cases number for each county and death number for each county.

### Methods

#### Spatial autocorrelation analysis

Spatial autocorrelation analysis is applied to describe the similarity of geographically proximate units. In this study, spatial autocorrelation analysis was used to clarify the overall autocorrelation pattern of HFRS incidence (clustered, dispersed, or random). Global Moran’s *I* statistic, developed by Patrick Alfred Pierce Moran [20], is one of the most commonly used methods in spatial autocorrelation analysis. It calculates the autocorrelation (*I*) as follows:

$$I=\frac{n}{\sum_{i=1}^{n}\sum_{j=1}^{n}\omega_{i,j}}\frac{\sum_{i=1}^{n}\sum_{j=1}^{n}\omega_{i,j}z_{i}z_{j}}{\sum_{i=1}^{n}z_{i}^{2}}$$ (1)

Where *n* is the total number of counties, x_*i*_ is the number of cases for county *i*, *X*_*j*_ is the number of cases for county *j*. *z*_*i*_ and *z*_*j*_ are the deviation values for county *i* and county *j* from their mean $\left(x_{i}-\bar{X}\right)$ and $\left(x_{j}-\bar{X}\right)$ respectively. *ω*_*i*,*j*_ is the spatial weight which used to quantify the spatial relationships between county *i* and county *j*. Spatial weight is structured into a matrix, the distance between county *i* and county *j* is calculated with *K* nearest neighbours.

Moran’s *I* index values for each year are calculated to measure spatial autocorrelation characteristics. Moran’s *I* index value ranges from −1.0 to +1.0. A zero Moran’s *I* Index value indicates a random spatial pattern [17]. Negative values indicate negative spatial autocorrelation while positive values indicate positive spatial autocorrelation.

Spatial autocorrelation analysis measures the globe spatial autocorrelation characteristics based on both county locations and number of HFRS cases simultaneously. It evaluates the spatial distribution pattern by a series number of county locations and the associated number values of HFRS cases.

#### Spatio-temporal scan statistical analysis

It is helpful to identify the moving tendencies of HFRS incidence in Hubei Province by investigating its spatial and temporal clustering areas. After the first step of spatial autocorrelation analysis, if HFRS occurred in a clustered way (which can be treated as an epidemic), the spatio-temporal scan statistical analysis could be used to identify the spatial and temporal characteristics of this epidemic **[5,11,15,21,22]**. Kulldorff scan statistic method was widely used to measure the degree of clustering **[23–26]**. This method imposed a cylindrical window to detect spatio-temporal clusters by constantly changing the size of the window, it has a circular (or elliptic) geographic base and a height corresponding to the disease onset time **[14,20,27,28]**.

Using this method, the radius of the cylindrical window varies continuously from zero to a specified maximum size. The maximum-size specified the percentage of the maximum total population at risk within the scanning window [29]. In this research, considering the human population density and the size of each county, the maximum cluster size was set to 50%. Discrete Poisson Model was used to identify the cluster locations of HFRS cases in a specific window. Moreover, SaTScan program was used to implement the scan statistical analysis [29–32].

#### Spearman's rank correlation analysis

Previous studies have showed that the transmission of HFRS in Hubei/China was influenced by environmental factors such as temperature, humidity and rainfall **[10,26,33,34]**. Climate variables have some relationships with dynamic rodent population, which can be used as possible influencing factors for the prediction of the HFRS epidemic **[12]**. In order to better understand influencing factors of the epidemic HFRS incidence, average temperature, average humidity, total rainfall and human population density were studied in this research. Spearman's rank correlation coefficients were used to measure the associated degree between variables **[35,36]**.

## Results

### Features of HFRS outbreaks in Hubei Province

**Fig 2:**
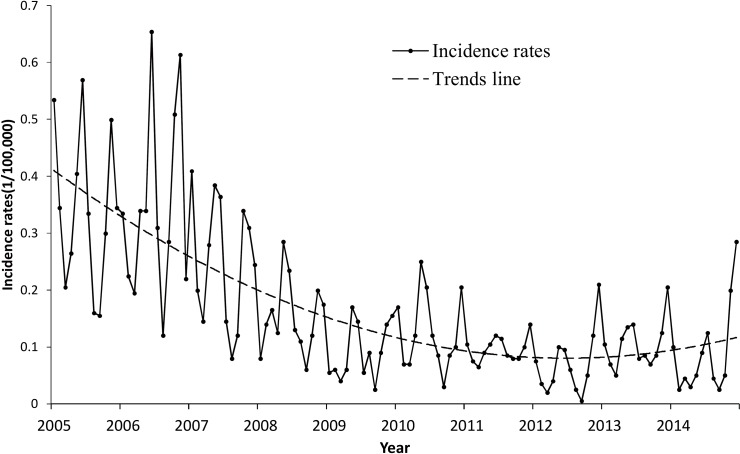
Incidence and trends in Hubei Province from 2005 to 2014. Each point represents the HFRS incidence rate in a specific year. All of the points are lined to indicate the trend of the HFRS incidence in Hubei Province. Trend line is simulated according to the incidence rates.

First, the ratio of HFRS cases in each month over the whole year, we define it as the HFRS outbreak value. Fig 2 demonstrates the HFRS outbreak rates for each month from 2005 to 2014 in Hubei Province. We observed the value of the rate fluctuated month by month, and had the maximum value of 0.6534 (June 2006) and the minimum value of 0.05 (September 2012). In general, the simulated trend line shows that the HFRS incidence rate had a decreasing tendency. It indicates that HFRS incidence decreased in general while increasing slightly from 2012 to 2014.

### Spatial distribution of HFRS cases

**Fig 3:**
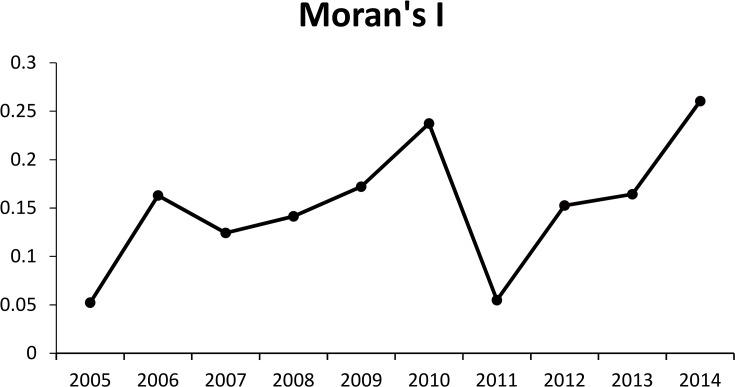
Distribution pattern of HFRS according to Moran's Index from 2005–2014. Each point represents the Moran’s *I* value in a specific year. All of the points are lined to indicate the trend of the Moran’s *I* in Hubei Province.

Fig 3 indicates the Moran’s *I* index values of the spatial autocorrelation results for HFRS cases from 2005 to 2014. Fig 3 and Table 1 indicate the spatial distribution of HFRS cases happened in 76 counties. Except 2005 and 2011, HFRS outbreak generally presented spatially clustered distribution with z-score>1.65 and p-value<0.05. The clustering degree of HFRS cases was gradually increased from 2006 to 2009 and reached its peak in 2010 (Moran’s I = 0.2374 with z-score = 4.1336 and p-value = 0.0001), while it happening in a high discrete pattern with the lowest point in 2011 (Moran’s I = 0.0550 with z-score = 1.2442 and p-value = 0.2134). Since 2012, it recovered to the clustering pattern till 2014 (Moran’s I = 0.2604 with z-score = 4.4143 and p-value = 0.0001).

**Table 1:** Spatial Autocorrelation results of average annual incidence rate of each year for HFRS in Hubei Province.

year	Moran's Index	Expected Index	Variance	z-score	p-value
2005	0.0523	-0.0133	0.0055	0.8844	0.3765
2006	0.1630	-0.0133	0.0050	2.4834	0.0130
2007	0.1242	0.1242	0.1242	2.2474	0.0246
2008	0.1413	-0.0133	0.0026	3.0412	0.0024
2009	0.1722	-0.0133	0.0045	2.7571	0.0058
2010	0.2374	-0.0133	0.0037	4.1336	0.0001
2011	0.0550	-0.0133	0.0030	1.2442	0.2134
2012	0.1526	-0.0133	0.0038	2.6845	0.0073
2013	0.1642	-0.0133	0.0027	3.4013	0.0007
2014	0.2604	-0.0133	0.0038	4.4143	0.0001

### Clustering pattern of HFRS cases

The spatio-temporal scan statistical analysis showed that HFRS was not randomly distributed in space during these ten years, which achieved the same conclusion as our previous studies did [37]. In Table 2, the number of clusters decreases from 5 to 2 from 2005 to 2009 while total cases of HFRS decreasing from 824 to 217. Although the variation from 2010 to 2014 is relatively small, the overall trend is still decreasing in terms of the spatio-temporal clusters of HFRS cases from 2005 to 2014.

**Table 2:** Spatio-temporal Clusters of HFRS cases from 2005–2014 in Hubei Province.

Time	Cluster Number	Total Cases
2005	5	824
2006	5	830
2007	3	605
2008	2	365
2009	2	217
2010	2	302
2011	3	232
2012	2	167
2013	2	253
2014	1	214

**Fig 4:**
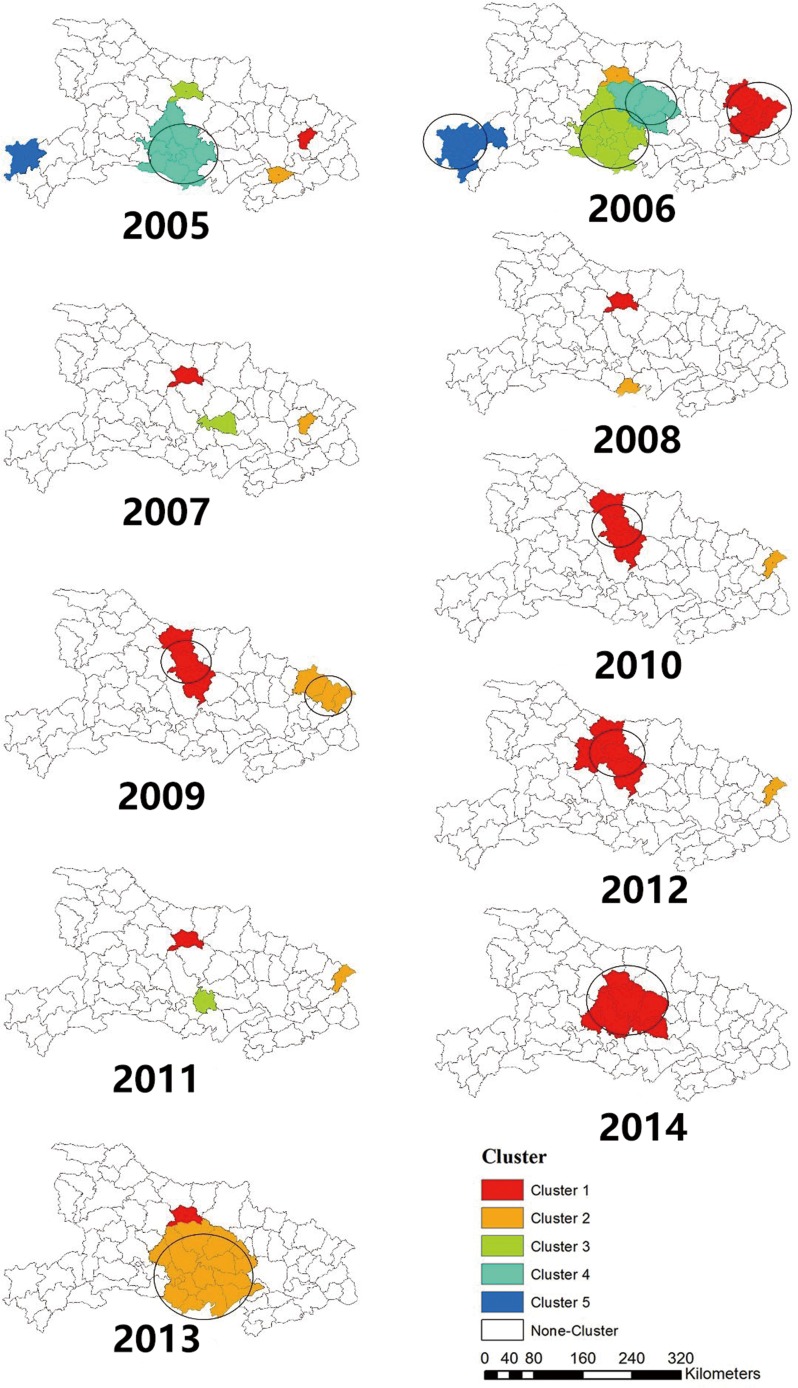
Clusters of HFRS cases from 2005 to 2014. Spatial and temporal clusters were identified each year from 2005 to 2014. Different colors represent different levels of clusters. The cluster one for each year was regarded as the Most Likely Cluster.

Fig 4 illustrates the exact locations of the clusters area and clustering levels. The Most Likely Cluster locates in cluster one. The clustering level decreases from cluster one (in red) to cluster five (in blue). The clusters distributed in various counties from west to east in 2005 and 2007. From 2007 to 2012, the clusters gradually moved towards the central area of Hubei Province. In 2014, only one cluster existed in the central area.

Table 3 represents the Most Likely Clusters of HFRS cases from 2005–2014 in Hubei Province, from Table 3, we can find that HFRS cases mostly occurred in the second half of the year (from summer to autumn during 2005, 2006, 2007, 2009, 2012, 2013 and 2014). Further, the number of the counties included in cluster one varied from 2005 to 2014. For example, cluster one includes only one county (Huang Gang) in 2005 but 5 counties (Macheng, Luotian, Yingshan, Huanggang and Zuoshui) in 2006. Interesting, Yicheng County, as a special epidemic area, has been included in cluster one all the time from 2007 to 2014.

**Table 3:** The Most Likely Clusters (Cluster one in Fig 4) of HFRS cases from 2005–2014 in Hubei Province.

Year	StartDate	EndDate	Counties	P-value
2005	2005/6/1	2005/11/30	Huanggang	<0.01
2006	2006/6/1	2006/11/30	Macheng, Luotian,Yingshan, Huanggang, Zuoshui	<0.01
2007	2007/7/1	2007/12/31	Yicheng	<0.01
2008	2008/1/1	2008/6/30	Yicheng	<0.01
2009	2009/7/1	2009/12/31	Yicheng,Xiangfan,Zhongxiang	<0.01
2010	2010/4/1	2010/8/31	Yicheng,Xiangfan,Zhongxiang,	<0.01
2011	2011/1/1	2011/6/30	Yicheng	<0.01
2012	2012/10/1	2012/12/31	Yicheng, Xiangfan, Zhongxiang, Nanzhang	<0.01
2013	2013/7/1	2013/12/31	Yicheng	<0.01
2014	2014/7/1	2014/12/31	Yicheng,Zhongxiang, Jingshan, Dangyang, Jingmen, Tianmen	<0.01

### Influencing factors of HFRS cases

**Fig 5:**
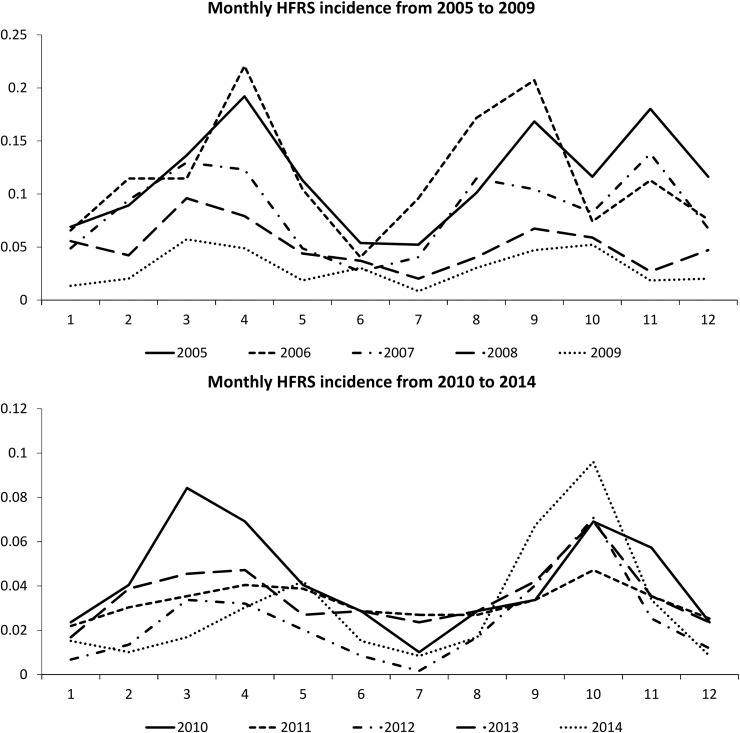
Monthly HFRS cases from 2005 to 2014. (A) Average monthly HRFS cases from 2005 to 2009. (B) Average monthly HRFS cases from 2009 to 2014.

Monthly HFRS outbreaks cases are presented in Fig 5 to explore the seasonal variation of HFRS outbreaks. Bimodal pattern is obviously observed noted that two-peak points appeared each year, one is in spring and the other is in autumn. In 2005, 2006 and 2007, one more peak points appeared during November.

**Fig 6:**
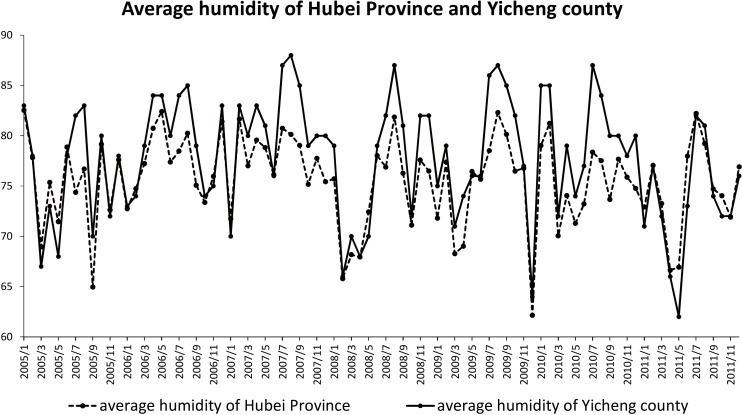
Comparison of the humidity of Yicheng County with the average humidity of Hubei Province. Average monthly humidity values for Hubei Province and Yicheng County are depicted by dotted line and solid line, respectively.

Related studies have demonstrated that climate factors had a great contribution to the outbreak of HFRS [3,7,10,12,38]. From Table 4, it seems that the average humidity influencing factor shows positive associated with HFRS cases (Spearman Correlation = 0.041 at the 0.001 level). Taking Yicheng County as an example, the value of its humidity is much higher than the average humidity level in the second half year (Fig 6). Combining the result of clusters in Fig 4 and the Most Likely Clusters in Table 3, results show that high humidity associate with the HFRS outbreaks consistently in Yicheng County.

**Table 4:** Correlation results for HFRS cases from 2005–2014 in Hubei Province.

Factors	Spearman Correlation	Sig. (2-tailed)
average temperature	0.011	0.417
average humidity	0.041**	0.001
total rainfall	0.015	0.234
human population density	0.397**	0.000

**. Correlation is significant at the 0.001 level (2-tailed).

HFRS is an infectious disease. The related studies have indicated that the density of the virus carriers, human immunity and immigration played significant roles in the transmission of HFRS [10,39]. In Table 4, the correlation analysis result, which values are with Spearman Correlation = 0.397 and with p-value≤0.001, means that the human population density factor is positive associate with the outbreak of HFRS epidemic in Hubei Province.

## Discussion

In this study, the relationships between the HFRS cases, spatio-temporal distribution and clustering patterns have been studied. We also explored some influencing factors for the HFRS cases in Hubei Province.

Our previous study of the past three decades of HFRS fatalities data revealed a general trend of decreased in Hubei Province [40]. No further outbreaks of HFRS occurred since 2000 after the two peaks happened in the 1980s and the in the 1990s, respectively [37]. In this study, we found the HFRS case increased from 2012 to 2014 while the general trend showed the HFRS cases decreased from 2005 to 2012. This phenomenon could possibly be explained the following thirdly factors.

First, *Hantaviruses* were carried and transmitted by rodents [39]. Xiao et al. found that rodent population density and the types of the rodent species significantly impact on the occurrence of HFRS [41]. An experiment from State Key Laboratory of Virology, Institute of Medical Virology, School of Medicine in Wuhan University, China has shown that the mice positive rate dropped from 9.16% to 0.28% from 2005 to 2011. But in 2012 the rate suddenly rose to 19%. The more frequently the aerosolized urine, droppings or saliva of infected rodents people touched, the higher infection possibility people will have. In addition, individuals working with living rodents could get a higher probability of infection. The effect of a rapidly growing rodent population on the environmental load of *hantaviruses* has been studied by Sauvage et al. [42] Their results demonstrated that the increasing of the rodent population and the concomitant in the environmental contamination is linked to an increasing risk of human infection. In this study, we inferred that the increase of HFRS incidence in Hubei Province from 2012 might be caused by the suddenly increasing of infected rodent population.

**Fig 7:**
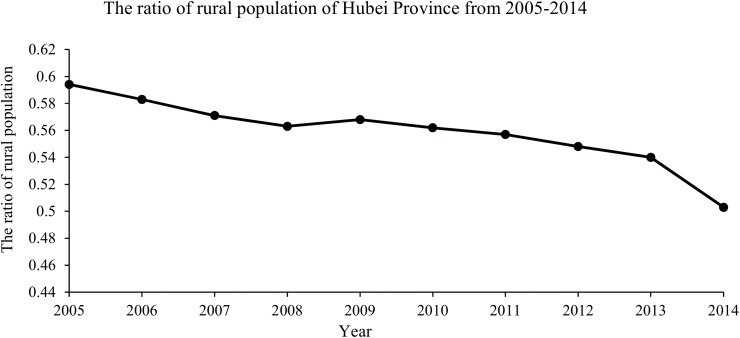
The ratio of rural population over the total population of Hubei Province from 2005–2014. Each point represents the ratio in a specific year. All of the points are lined to indicate the trend of the rural population ratio in Hubei Province from 2005 to 2014.

Secondly, human migration (from rural areas to cities) and urbanization have both reduced the risk of exposing to rodent excreta. As shown in Fig 7, the ratio of rural population over total population in Hubei Province is decreasing year-by-year. It may affect the incidence of HFRS as the decreased trend in the whole period.

**Fig 8:**
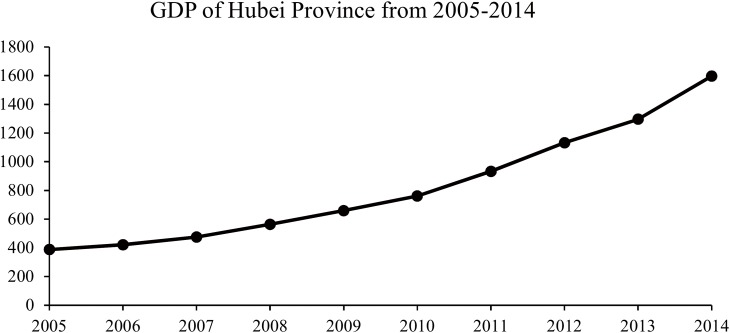
GDP of Hubei Province from 2005–2014. Each point represents the GDP value in a specific year.

Thirdly, Fig 8 shows the continuous rising of GDP in Hubei Province from 2005 to 2014. The growth of GDP improves housing conditions and workplace conditions. These will be beneficial to the reduce of rodent-to-human contact. According to the statistical data acquired from Hubei Provincial Bureau of Statistics, medical and health institutions in Hubei Province increased from 9983 to 36089 during 2005–2014.

In the study, we also found that the clustering degree was increasing and gathering to the central area of Hubei Province. In addition, from geographic characteristics perspective, HFRS has not been endemic near abundant water resources until 2005. Combining with the topography, geomorphology and vegetation covered environments, this finding might explain by the following.

The previous studies have demonstrated that HFRS in Hubei Province was mainly affected by SEOV virus which was transmitted by Rattus norvegicus during 1980–2012 [37,39]. Hubei Province is surrounded on three sides (east, west and north) by mountains. Its low and flat middle part is a piece of incomplete basin slightly open towards south. Counties located in the central part possess the subtropical humid monsoon and monsoon climate. This can provide a better living environment for the survival and reproduction of the rodents (like Rattus norvegicus), and the Rattus norvegicus could easily carry SEOV virus.

The industrial development often means high-speed economy development and the concentration of population. As listed in Table 3, Yicheng County appeared in the Most Likely Clusters consistently. Industry and agriculture data, collected from Hubei Provincial Bureau of Statistics, indicated that the industrial production from 2007 to 2014 increased from 3.001 billion to 16.717 billion by 457% percentage in Yicheng County. While for the none cluster area like Shennongjia County, for example, the number of industrial production from 2007 to 2014 increased from 42.062 million to 75.322 billion by only 79% percentage. Compared with the high human population density of 192 per square kilometre for Yicheng County, the population density in Shennongjia County only covers 18 per square kilometre. We believe that the industrial development and human population density may affect the number of HFRS cases.

**Fig 9:**
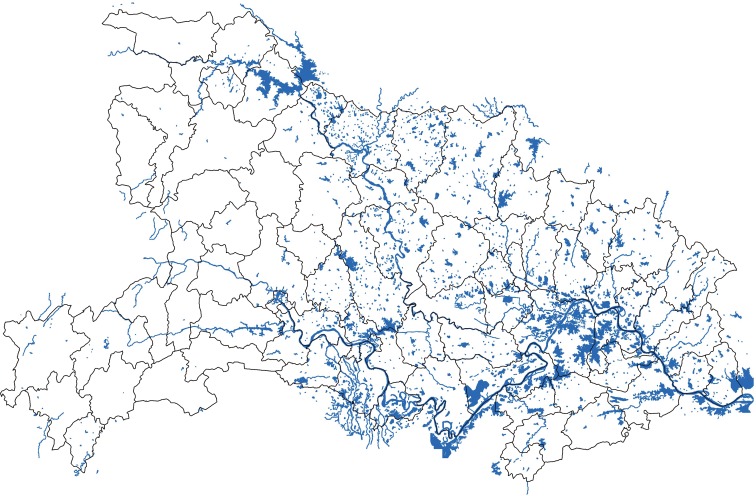
Water distribution in Hubei Province. The colored areas indicate the water boundaries in Hubei Province.

**Fig 10:**
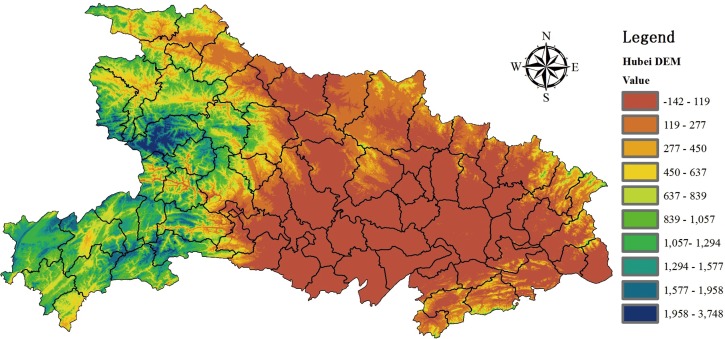
Digital Elevation Model(DEM) Data for Hubei Province. The color gradient presents the elevation value of Hubei Province in thematic maps. It is grouped in 10 categories based on the elevation value for each location.

Peng et al. found that HFRS cases were distributed along large water systems (wetland) between 1983 and 1995, such as Yangtze river and Huai river in Anhui Province [26]. The surrounding water system is helpful to the cultivation of crops. It will also create a better environment for rodents to survive and thrive. In Fig 9, the blue area represents water system that mainly distributes in the east part. In Fig 4, the cluster gathering area is in the western region during 2005 and 2006. And then, a cluster gathering area mainly concentrated in the northern central area in the following years (2007–2014). As a result, the cluster aggregation of HFRS has not showed a significant correlation with the water system. For instance, Yicheng County has about 115 square kilometres water area, the average annual HFRS cases rate during this period is 8.11. However, Wuhan City, Honghu City and Danjiangkou City have a water area of 1040, 622 and 400 square kilometres respectively, which rank among the top three largest water area cities. The HFRS cases rate corresponding to the three cities are only 0.13,0.77 and 0. In Fig 10, elevation values from DEM data are presented. The central, southern and eastern region are mainly plain areas or small hilly areas with relatively low elevation values (their average elevation is between -142 to 277 meters). Combined with Fig 4, HFRS case clusters mostly gathered in the central and southern part. One possible explanation may be that HFRS outbreak more easily in plains rather than in mountains.

As displayed in Fig 5, the number of HFRS cases shows that there are two peaks annually. One happens in spring time (March to May). It is corresponded to the epidemic peak of SEOV-type HFRS emerged in spring time. Another peak appears between September and November, the peak of HTNV-type HFRS was often emerged during these time period [39,43]. This phenomenon indicates that the causes of HFRS in Hubei Province might be associated with both types of virus (HTNV and SEOV). Compared with the peaks happened in each year, and data revealed from Fig 5, we could infer that if peaks mostly emerged in spring time during a year, it could be presumed that SEOV-type HFRS have more influences vice versa.

Pervious study has concluded that there are many environmental factors that impact the spatio-temporal dynamics of HFRS, such as the changes of precipitation, temperature, land use and vegetation community dynamics[44]. For example, temperatures may affect the dynamics and activities of rodent hosts as well as the infectivity of *hantavirus* [40]. In this research, it was demonstrated that the factors of human population density and average humidity are significantly related to HFRS epidemic in Hubei Province.

Li et al. found that the increase of human population density could make the transmission of the virus much easier [43]. As shown in Table 4, human population density factor is positively associated with the outbreaks of HFRS. It can be inferred that the congregation of population may provide more opportunities for the transmission of HFRS virus.

Climate factors have been long considered as important impact factors for the spreading of HFRS [10,12,26]. In our research, average humidity was positively associated with the outbreak of HFRS. Other environmental factors (such as average temperature, total rainfall) did not display significant relations with the outbreak of HFRS. The result was different from the other researches. One possible explanation is that we used data from different regions. For example, Liu et al. believed that rainfall, land surface temperature and relative humidity were significantly associated with monthly HFRS cases in Elunchun and Molidawahaner counties (China) for 1997–2007 [12]. However, Hubei Province is categorized as subtropical humid climate. It means the average humidity in Hubei Province is higher than the north part of China. Hubei Province is known as the "land of a thousand lakes". The climate and ecological conditions in Hubei Province provide an ideal habitat for rodents [45]. Xiao et al. demonstrated that moist environments are help for the vitality and infectivity of Hantaan virus and the distribution of rodents [41]. The humidity factor may affect several biological traits of the rodent hosts, from individual life histories to population dynamics. Corresponded with the correlation results from this research, it could be inferred that humid climate may contribute to the survival and reproduction for the virus. And this may contribute to the outbreaks of HFRS.

## Conclusions

The findings in our research presented some new features regarding the trend and influencing factors for the HFRS outbreaks in Hubei Province. First, SEOV and HTNV are both associated the HFRS outbreaks. HFRS seasonal distribution in Hubei Province was characterized by a bimodal pattern (March to May, September to November). This bimodal pattern was corresponding to the peaks of SEOV and HTNV virus respectively. Compared with the peaks happened in each year, we could infer that if peaks mostly emerged in spring time during a year, it could be presumed that SEOV-type HFRS have more influences vice versa. Secondly, the average humidity and human population density were associated with the HFRS epidemic. It can be inferred that the congregation of population may provide more opportunities for the transmission of HFRS virus. And humid climate may contribute to the survival and reproduction for the virus. Thirdly, HFRS outbreaks were more in plains than in other areas of Hubei Province. HFRS case clusters mostly gathered in the central and southern part, the central and southern regions are mainly plain areas or small hilly areas. We did not find that whether the terrain of the wetland (water system) plays a significant role in the outbreak of HFRS incidence. We believe these findings can better understand the spatio-temporal pattern and influencing factors, and can further help to improve the reducing cases of HFRS in the future.

## Supporting Information

S1 File Population Data of Hubei Province in 2005 (RAR)

S2 File Map at a scale of 1:4000,000 of Hubei Province (RAR)
